# Validation and human factor analysis study of an infant weight estimation device

**DOI:** 10.1186/s12887-020-1933-5

**Published:** 2020-01-22

**Authors:** Susan M. Abdel-Rahman, Ian M. Paul, Paula Delmore, Jia-Yuh Chen, Mary Mills, Rachel G. Greenberg, on behalf of the Best Pharmaceuticals for Children Act – Pediatric Trials Network

**Affiliations:** 1 Children’s Mercy, University of Missouri Kansas City-School of Medicine, Kansas City, MO USA; 2 Division of Clinical Pharmacology, Toxicology, and Therapeutic Innovation, 2401 Gillham Road, POB 2M02.47, Kansas City, MO 64108 USA; 3 Penn State College of Medicine, Hershey, PA USA; 4 Wesley Medical Center, Wichita, KS USA; 5 The Emmes Corporation, Rockville, MD USA; 6 Duke Clinical Research Institute, Durham, NC USA

**Keywords:** Chest circumference, Head circumference, Preterm, Full-term

## Abstract

**Background:**

Weight is critical for the medical management of infants; however, scales can be unavailable or inaccessible in some practice settings. We recently developed and validated a robust infant weight estimation method based on chest circumference (CC) and head circumference (HC). This study was designed to determine the human factors (HF) experience with, and predictive performance of, an infant weight estimation device that implements this method.

**Methods:**

Prospective, multi-center, observational, masked study of 486 preterm and term infants (0–90 days) assessed by 15 raters. Raters measured the infant using calibrated scales/measures and masked versions of the device. Raters also evaluated critical tasks associated with device use. Mean error (ME) and mean percentage error (MPE) were used to assess predictive performance.

**Result:**

Among 486 infants enrolled (36.8 ± 4.0 weeks gestational age, 31.5 ± 28.6 days postnatal age), predicted weight correlated highly with actual weight (r = 0.97, ME: − 69 ± 257 g, MPE: − 1.3 ± 6.9%). Predicted weight was within 10 and 15% of actual weight in 86 and 99%, of infants. HF errors were low, 0.1–0.8% depending on task. In all cases raters were confident or very confident in their measurements.

**Conclusion:**

The device was statistically equivalent to the method on which it was based and approximated weight with acceptable variance from the true weight. HF data suggest the device is easy to use. This device can be used to estimate weight in infants when calibrated scales are impractical or unavailable.

## Background

Body weight is the foremost marker of health and health outcomes during infancy. It is essential for evaluating growth and development and is a critical factor in safe and effective medical management throughout childhood [1–3]. A calibrated weighing scale remains the universal gold standard for obtaining weight in children; however weighing scales are often not available in resource-constrained settings across the globe [4–8]. Where scales are available, it can still be challenging to remove or account for the weight of life-sustaining medical equipment prior to obtaining a scale-based weight. Numerous proxies for weight in the newborn have been investigated; however, the majority of studies are designed to identify thresholds that discriminate low birth weight newborns [9]. In response to the need for a simple, robust weight estimation strategy, we developed a method for estimating weight in infants from the circumference of the head and chest [10]. The method was based on a similar approach developed previously for weight estimation in children [11], and the resultant prototype (Mercy babyTAPE) is a variation on the U.S. Food and Drug Administration cleared pediatric device (MercyTAPE). (https://www.accessdata.fda.gov/scripts/cdrh/cfdocs/cfpmn/pmn.cfm?ID=K142469).

The final element of a risk management process designed to reduce design-related problems that contribute to unsafe or ineffective use of a medical device is human factors validation testing. For devices that represent a modification of a device already on the market, the risk analysis is expected to focus on aspects of the device that were affected by the modification including the users’ interactions with the device [12]. The primary objectives of our study were to examine the critical tasks for the safe and effective operation of the babyTAPE in a device use environment and confirm the predictive performance of the babyTAPE when applied by end-users. Secondary aims of the study were to define inter-rater reliability for the babyTAPE and capture users’ subjective experience with the device.

## Materials/subjects and methods

### Study design

This was a prospective, multi-center, observational study conducted in the outpatient clinics and inpatient newborn units at three U.S. hospitals (Children’s Mercy, Kansas City, MO; Hershey Medical Center, Hershey, PA; Wesley Medical Center, Wichita, KS).

### Device

**Fig. 1:**
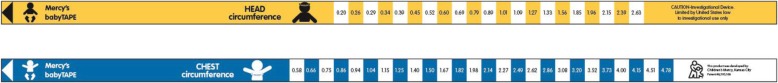
Mercy babyTAPE (not drawn to scale)

Details surrounding the method on which the babyTAPE device (i.e. the study device) is based have been previously published [10]. The babyTAPE device is a flexible, paper-based strip printed on both sides with one side (yellow) designated for head circumference measurements and the other (blue) designated for chest circumference measurement (Fig. 1). The “start” end on each side of the device is marked with a large contrasting triangle. Along the length of the babyTAPE are 1 cm (cm) “bins” of alternating color that have additional markings which correspond to fractional weight values. The estimated weight of the infant (in hundredths of a kilogram) is obtained by summing the fractional weights derived from the two measurements. Since raters had knowledge of the infant’s weight, a masked version of the device replaced fractional weight values with arbitrary alphanumeric characters to minimize the potential for bias. The characters were organized so that we could discern whether the correct side and the correct starting end of the investigational device were used to perform the measurements. The code was broken only after enrollment closed at all participating sites. Study devices were printed on paper, and checked against a National Institute of Standards and Technology (NIST)-certified ruler in compliance with International Organization for Standardization (ISO) 9000 standards.

### Participant infants

All infants presenting to the participating institutions of any gestational age who were 0–90 days of life were eligible for enrollment. Infants were stratified into 9 postmenstrual age blocks to ensure balanced enrollment and an even distribution of participants across weight and length. Participants were excluded if there were known or apparent anatomical deformities, external medical equipment that would impair the determination of actual weight, if they were incapable of having the measurements performed, or if the investigator or treating physician perceived contraindications to their inclusion. All infants were enrolled with informed parental permission under a protocol that was reviewed and approved by the Institutional Review Boards of the respective study sites.

### Participant raters

Study raters were required to qualify for participation by demonstrating accuracy and reproducibility measuring head- and chest-circumference. Prospective raters made three sets of non-sequential measurements on three infant sized mannequins using a standard tape measure and the babyTAPE. Pre-study training was provided to ensure that the raters could identify the correct anatomic landmarks and read a standard tape measure. However, specific training on application of the babyTAPE was not provided. Rather, raters were provided with the babyTAPE “Instructions for Use” (Additional file 1) and evaluated according to their ability to execute the measurements based on these instructions. Intra-rater variance was calculated for each rater and could not exceed 5% for any measurement. Raters that failed qualification were remediated and given the opportunity to repeat the qualification. Raters that failed the second qualification were not be permitted to participate in the study.

### Measurements

At enrollment we recorded participants’ gestational age, postnatal age, sex, race, and ethnicity. Weight was determined using a calibrated infant scale after removing clothing and diapers. Length was obtained using standard medical equipment available in patient care areas of the participating institution. Circumferential measures (in millimeters) were performed with a standard vinyl tape measure that was checked against an NIST-certified ruler. Chest circumference was determined with the infant’s arms extended outward to shoulder level and the tape measure placed under the axilla and around the chest, passing by the xyphoid process at the level of the nipple. Every effort was made to record chest circumference at the end of exhalation. To obtain head circumference, the tape measure was placed around the infant’s head so that it lay across the frontal bones, slightly above the eyebrows and ears, over the occipital prominence at the back of the head, perpendicular to the long axis of the face. The same technique for measuring head and chest circumference was applied for both the standard tape measure and the babyTAPE. All measurements were performed at the same time by a single rater. Approximately 10% of participants at each site were selected for multi-rater assessment to examine inter-rater reliability. After completion of measurements, infants were observed for an additional 10 min to assess study-related adverse device effects (ADEs).

After performing all study-related measures, subjective assessments of the babyTAPE were provided by each rater for each enrolled child via the following questions: *Did you have any trouble identifying the proper landmarks on the infant? Could you correctly identify the proper starting ends of the babyTAPE for the measurements? Did you experience any difficulty performing the measurements on this infant with the babyTAPE?* *Were the circumference markings on the babyTAPE easy for you to read?* *Using the same babyTAPE you just used, how confident are you that you would obtain the same readings if you repeated them right now on the same infant?*

### Data analysis

Three critical tasks were defined a priori: 1) identification of the correct anatomic landmarks, 2) proper use and orientation of the device, and 3) accurate observation and recording of the device outputs. Critical task #1 was examined by determining whether recorded chest and head circumferences measured using the babyTAPE were within the expected range for each participant’s age and weight. Chest circumference measurements were compared to reference data collected in a published anthropometric study [13]. Head circumference measurements were compared to the Center for Disease Control and Prevention-National Health and Nutrition Examination Survey reference data [14]. Absolute z-scores of > 3 were classified as extreme outliers and examined for a possible misidentification of anatomical landmarks in conjunction with the infants z-score for length and weight.

Critical task #2 was examined by determining whether measurements recorded for the babyTAPE and reference tape were concordant. Reference tape measurements were binned to the nearest centimeter before evaluation. Discrepancies of ≥3 bins were identified as indicative of errors with the measurement and/or recording of reference tape or babyTAPE-values. In these cases, the estimated weight assigned by each device was evaluated in an attempt to identify the clinical significance of the erroneous measurement.

Critical task #3 was examined by determining whether recorded values for babyTAPE circumferences measurements appeared on the device as printed and appeared on the side of the device indicated by the user. The number and percentage of measurements with observation or recording errors were summarized.

The predictive performance of the babyTAPE was established by comparing the babyTAPE predicted weights with weights measured on a calibrated medical scale. The difference between these measurements were summarized using statistics that include the mean error, mean squared error, and proportion within 10 and 15% of actual weight. Parameter estimates and 95% confidence intervals were compared with results found from the validation study of the method that the babyTAPE embodies.

Between-user variability was examined by comparing the babyTAPE and reference tape circumference measures from the multi-rater assessment. Between-user variability was characterized through estimation of the intraclass correlation coefficient (ICC), the accompanying 95% confidence intervals, and the proportion of observations ≥10% apart. Agreement between estimated weight and actual weight was determined using Bland-Altman plots with log-transformation. IBM SPSS version 24 (IBM Corp., Armonk, NY) and SAS (version 9.3, SAS Institute, Inc., Cary, NC) were used for all analyses.

### Sample size calculation

Sample size was estimated based on the ability to discriminate device-estimated weight from actual weight. Assuming an observed proportion of estimated weights that differ from actual weight by ≤10% of 0.8, a two-sided 95% Wilson’s score confidence interval is (0.76, 0.83) with 460 participants after accounting for drop-outs. Additionally, selection of at least 50 participants for multi-rater assessment provides > 80% power to conclude that the ICC is > 0.8 when the true ICC is 0.9.

## Results

**Fig. 2:**
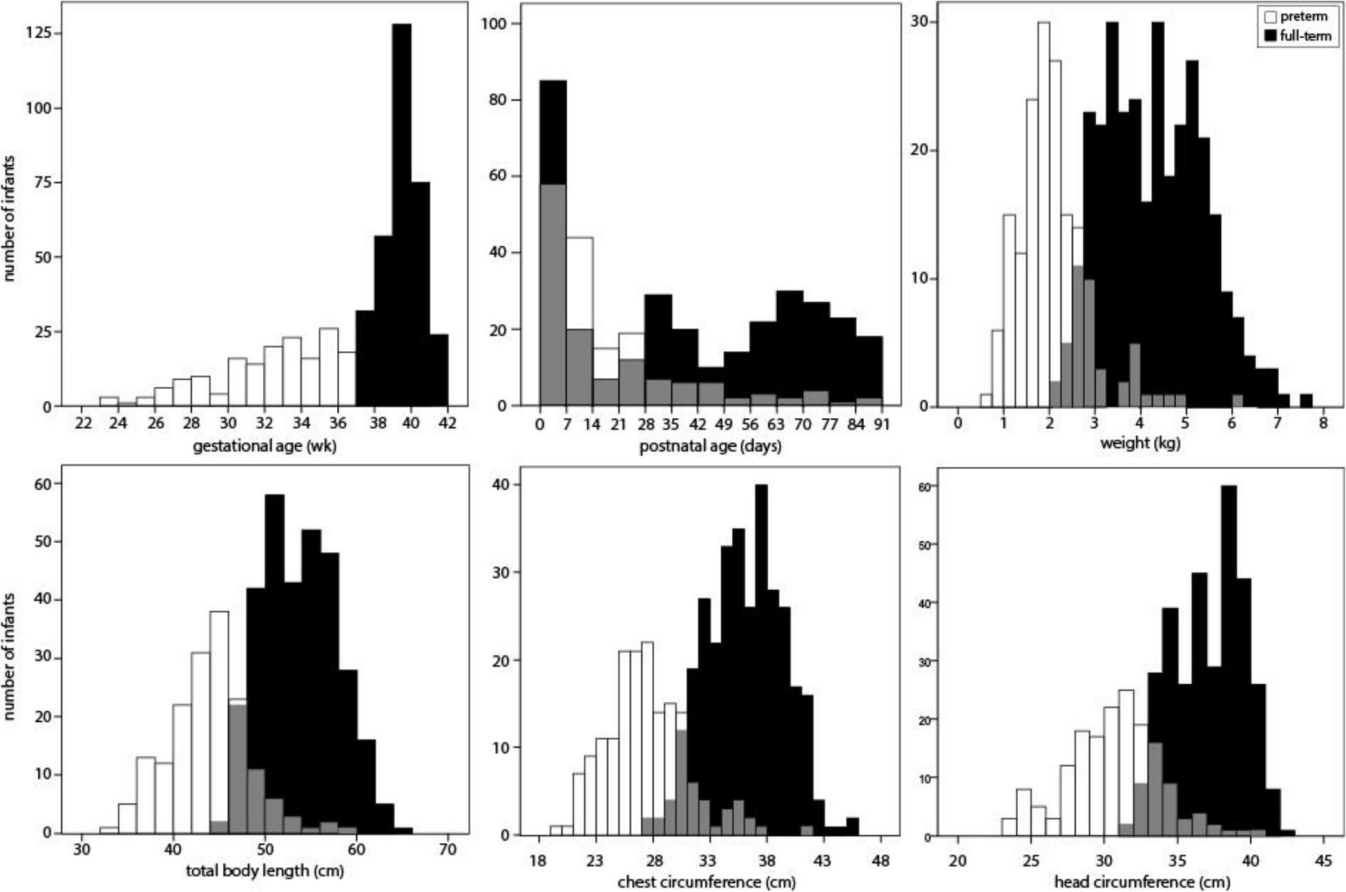
Histograms depicting the study population distribution for gestational age (left), postnatal age (center), and weight (right). Grey areas depict overlap of preterm and full-term infants

In total, 486 participants were enrolled across the three clinical study sites. For these infants, gestational age averaged 36.8 ± 4.0 weeks and postnatal age 31.5 ± 28.6 days (Fig. 2). Participants were 52% male; 62% were white, 24% were black, and 22% were Hispanic or Latino. The distribution of weight across the population ranged from 682 to 7590 g as depicted in Fig. 2 along with the distribution of the other anthropometric parameters. With respect to habitus, weight-for-length z-scores ranged from − 3.5 to 3.7 with 133 infants under the 5th %tile, 300 between the 5th and 84th %tile, 39 between the 85th and 95th %ile, and 14 above the 95th %ile. Seventeen raters participated in the training; 15 who passed on their first attempt and 2 who passed after remediation; however, only 15 participated in the study. Across the study, no ADEs were reported.

No child was excluded for anthropometric measurements outside of the expected range for age and gender (Critical task #1). For the objective determination of the potential for human error with this device, measurement differences of > 3 cm between the babyTAPE and reference tape were examined. The data revealed that 0.6% of the 972 total babyTAPE measurements were discordant with the measurements made using the reference tape (Critical task #2). These were comprised of 6 measurements in 4 children performed by 3 raters (Table 1). In each case, the error (as reflected by the impact on estimated weight) appears to have been with the reference tape measure rather than the babyTAPE device. Application of the weight estimation method using a reference tape measure resulted in percentage error ranging from − 12.2 to 18.4% and absolute percent error greater than or equal to 5% in all participants. In contrast, weight estimation by the babyTAPE in these infants resulted in percentage error ranging from − 1.4 to 2% and absolute percent error ≤ 2% in all participants. In no case was the incorrect side or incorrect end of the device used (Critical task #3).

**Table 1:** Measurement errors uncovered as part of the human factors analysis in 486 infants

Rater	Discordant Measure	EGA	PNA	Actual weight (kg)	Reference est. wt. (kg)	Reference error (%)	babyTAPE est. wt. (kg)	babyTAPE error (%)
1	CC, HC	38 wk., 3 d	19 d	3.335	3.75	12.4%	3.31	−0.75%
4	CC, HC	40 wk., 0 d	53 d	3.970	4.17	5.0%	4.05	2.0%
12	HC	41 wk., 3 d	70 d	5.526	4.85	−12.2%	5.48	−0.8%
12	CC	41 wk., 1 d	29 d	4.534	5.37	18.4%	4.47	−1.4%

*CC* Chest circumference, *d* days, *EGA* Estimated gestational age, *est. wt*. estimated weight, *HC* Head circumference, *kg* kilograms, *PNA* Postnatal age, *wk* weeks

A total of 36 participants were selected for multi-rater measures. The ICC for chest circumference and head circumference measured using the babyTAPE were 1.00 (1.00, 1.00). For measurements made with a reference tape measure the ICC for chest circumference was 0.99 (0.99, 1.00) and head circumference was 1.00 (0.99, 1.00). In no case was the auditor-derived measurement ≥10% different from the rater-derived measurements. For head circumference, rater and auditor measures differed in the range of − 0.5 to 0.6 cm (− 1.69 to 1.74%). Chest circumference measures varied from − 1.6 to 1.4 cm (− 4.42 to 4.38%).

Subjective perceptions of the critical human factors tasks are described as follows. Of 972 total measurements, only one rater indicated difficulty in identifying the proper anatomic landmarks for the chest in one participant. No other raters expressed difficulty with identifying landmarks in the head and chest. In two and six cases, respectively (0.2 and 0.6% of measurements), raters indicated difficulty identifying the proper starting end of the device or reading the markings on the device. Finally, raters expressed difficulty making the chest measurement in five children and the head measurement in three children. Overall, 100% of raters were confident or very confident of obtaining the same readings if repeated immediately on the same infant.

**Fig. 3:**
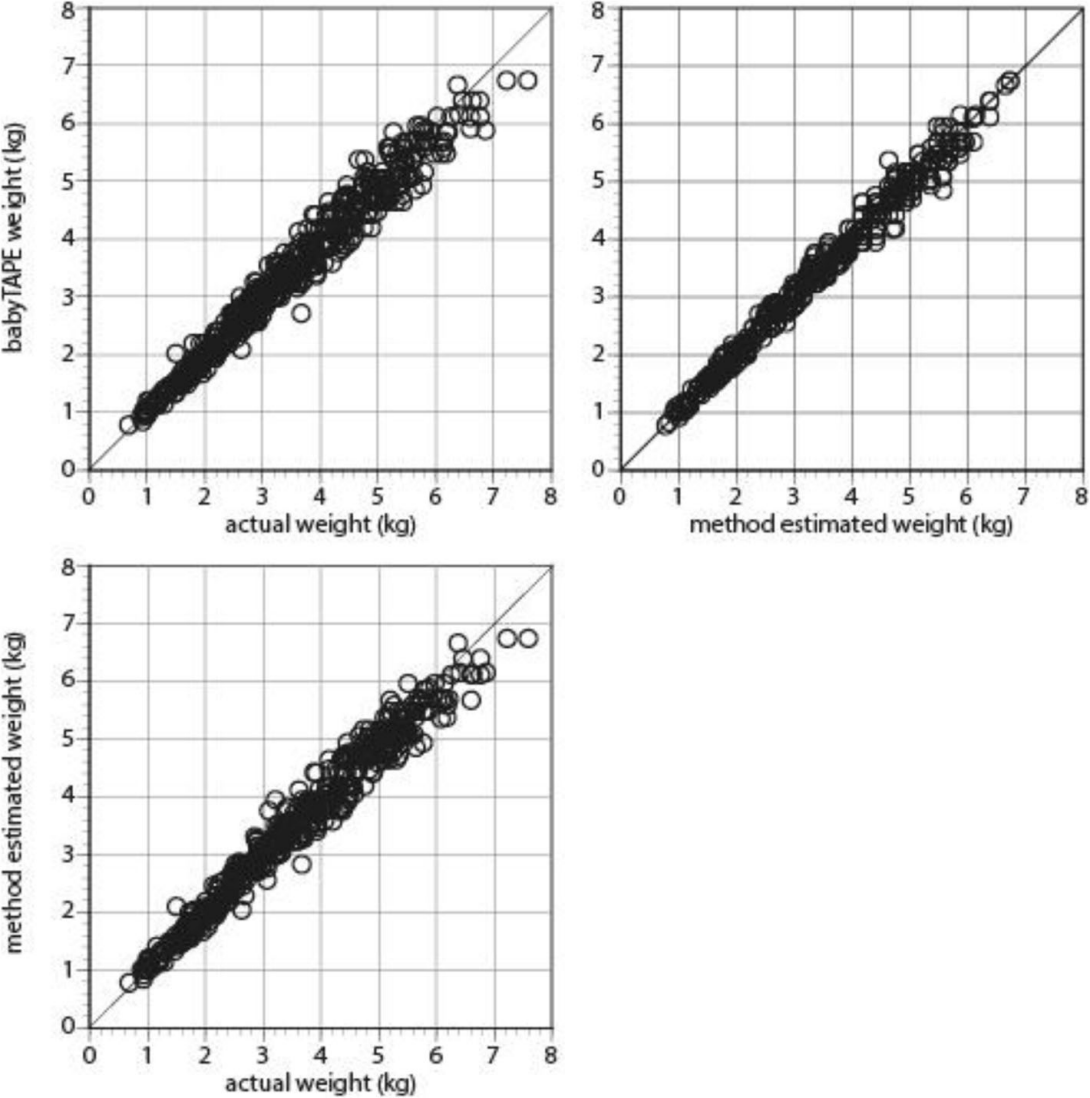
Scatterplot depicting the concordance between actual weight and device-estimated weight (upper) or method estimated weight (lower)

There was substantial agreement between the infants’ actual weight and the device predicted weight (Fig. 3, Additional file 2: Figure S1). The regression slope (confidence interval, CI) was 0.96 (95% CI 0.94, 0.98) with an intercept of 0.076 (95%CI 0.03, 0.12). Exploring the magnitude of this deviation reveals a mean error of − 69 ± 257 g corresponding to a mean percentage error of − 1.3 ± 6.9%. There was also strong agreement between the infants’ actual weight and the method from which the babyTAPE device was developed (Fig. 3, Additional file 2: Figure S1). For this regression the slope was 0.95 (95% CI 0.94, 0.97), the intercept 0.113 (95%CI 0.07, 0.16), the mean error − 66 ± 249 g, and the mean percentage error − 1.1 ± 7.1%. The fraction of infants in whom the babyTAPE device predicted weight within 10 and 15% of actual weight was 0.86 and 0.99, respectively.

## Discussion

Several devices have been described for classifying newborns as “low birth weight” based on measures of chest circumference, thigh circumference, upper-arm circumference, or foot length [15–18]. To our knowledge, however, there are no devices that facilitate the estimation of kilogram weight in infants*.* In a previous study we developed a method for weight estimation in infants through the first 90 days of life [10]. In this study, a prototype device based on this method was applied by representative end-users to pediatric patients in a device use environment. In support of an anticipated regulatory submission for device clearance, the primary objective of this study was to establish whether the babyTAPE could be used safely and effectively in the intended population.

Overall the babyTAPE slightly under predicted weight as evidenced by a mean error, mean percentage error, and regression slope of − 69 g, − 1.3%, and 0.96, respectively. With respect to predictive performance, the babyTAPE appears to be statistically equivalent to the method on which it was based and the device approximates infant weights with acceptable variance from the true weight. With 86% of infants in this study predicted within 10% of actual and 99% predicted within 15% of actual, the babyTAPE is actually slightly more robust than the MercyTAPE evaluated using the same trial design, in which 76 and 98% of children were predicted within 10 and 20% of their actual weight, respectively [19]. This is likely explained because the babyTAPE was developed for a much narrower age band (0–90 days) than the predecessor device, which was designed for children 2 months to 16 years of age. We also speculate that the anatomic measures selected for the babyTAPE are easier for the users to perform. Though still reproducibly obtained, the ICC for measures used by the MercyTAPE, namely humeral length and mid-upper arm circumference, are slightly lower than observed for head circumference and chest circumference (0.94 [0.92,0.96] for both measures) [13].

Collectively, challenges experienced with any of the critical tasks (i.e. identification of the correct anatomic landmarks, proper use and orientation of the device, accurate observation and recording of the device outputs) occurred at a rate of < 1%. There were also no instances in which the user did not feel confident or highly confident with their measurements. These subjective perceptions imply that users find the babyTAPE easy to use. This is reflected in the objective data wherein we observed only 6 instances in 4 patients where the babyTAPE and the standard tape measure were inconsistently applied. Notably, in each of the 4 cases, the error appears to have been with the reference tape measure rather than the babyTAPE device. This is not the first time we have experienced an issue with health care providers having trouble using and/or accurately reading values from a tape measure [20, 21]. In fact, it is one factor that plays a role in the failure of raters to qualify for participation in our anthropometric studies. Consequently, the use of a binning strategy on the babyTAPE, where singular values span 1 cm blocks, may play a role in mitigating problems that arise when practitioners are trying to read a reference tape measure with discrete 1 mm intervals.

We would be remiss not to highlight the limitations of this study. Participating raters were required to demonstrate proficiency in performing head and chest circumference measurements. This may not reflect the skills of the provider using the device in a clinical setting and thus could overestimate performance of the device. However, none of the raters in this study represented neonatal clinicians who may have greater familiarity with these measurements in this population. Nevertheless, the accompanying instruction sheet was created in an attempt to provide the necessary education. The study also enrolled children who were stable enough to have measurements performed. As a result, we are unable to comment on the ability to use the device effectively in unstable, critically ill-infants. Finally, the number of children enrolled under 30 weeks of gestation was low and additional experience may be required to inform of usability challenges at the lower extreme of age.

## Conclusions

To our knowledge, the Mercy babyTAPE represents the first device that has been successfully validated for accurate weight estimation of preterm and term newborns. Though the end users will have to determine whether the predictive performance of the babyTAPE is suitable for their indication, the data presented herein support the assertion that the babyTAPE can be reliably, reproducibly, and comfortably used by trained providers to estimate weight in young infants when calibrated scales are unavailable.

## Supplementary information

**Additional file 1.** Mercy babyTAPE Instructions for Use.

**Additional file 2.** Supplemental Figure, Bland-Altman plots depicting the difference between device-predicted weight versus actual weight (upper), method-predicted weight versus actual weight (middle), and device-predicted weight versus method-predicted weight (lower). Dashed lines depict the 95% limits of agreement.

## Data Availability

The datasets used and/or analysed during the current study are available from the corresponding author on reasonable request.

## Abbreviations

ADE: Adverse device effects
CC: Chest circumference
cm: centimeter
d: days
EGA: Estimated gestational age
est. wt: estimated weight
HC: Head circumference
HF: Human factors
ICC: Intraclass correlation coefficient
ISO: International Organization for Standardization
kg: kilograms
ME: Mean error
MPE: Mean percentage error
NIST: National Institute of Standards and Technology
PNA: Postnatal age
wk: weeks
