# Supplementary material for: Validation and human factor analysis study of an infant weight estimation device
Source: BMC Pediatr. 2020 Jan 22;20:30. doi: 10.1186/s12887-020-1933-5 (PMC6977278; doi:10.1186/s12887-020-1933-5)
Supplement: Supplementary file 1 — Additional file 1. Mercy babyTAPE Instructions for Use. [file 12887_2020_1933_MOESM1_ESM.pdf]

Mercy babyTAPE - Instructions for Use

A. Estimating Weight with the Mercy babyTAPE

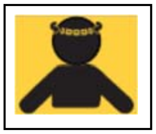

Head Circumference

– Place the TAPE around the infant's head so that it lies across the frontal bones, slightly above the eyebrows and ears, over the occipital prominence at the back of the head, perpendicular to the long axis of the face.

– Tighten the TAPE so that it fits snugly around the head and compresses the hair and underlying soft tissues and identify the square that spans the tip of the TAPE.

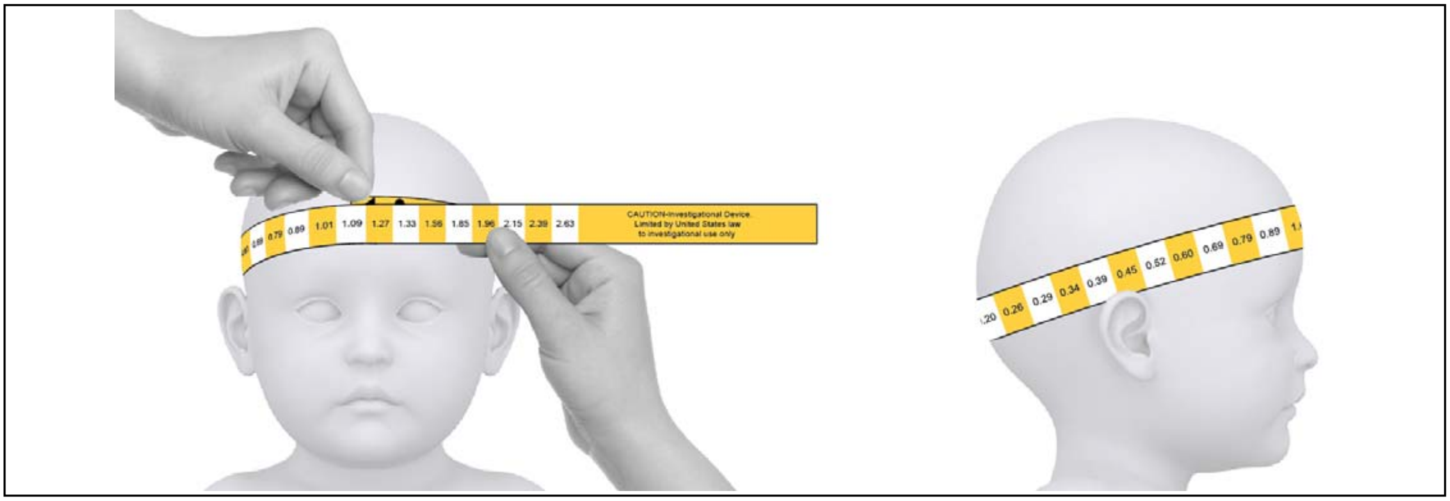

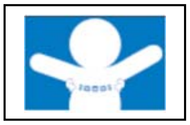

Chest Circumference

– Extend the infant's arms.

– Place the TAPE around their upper torso under their axilla and around the chest, passing by the xyphoid process at the level of the nipple.

– Tighten the TAPE so that it fits snugly around the chest and identify the square that spans the tip of the TAPE at the end of exhalation.

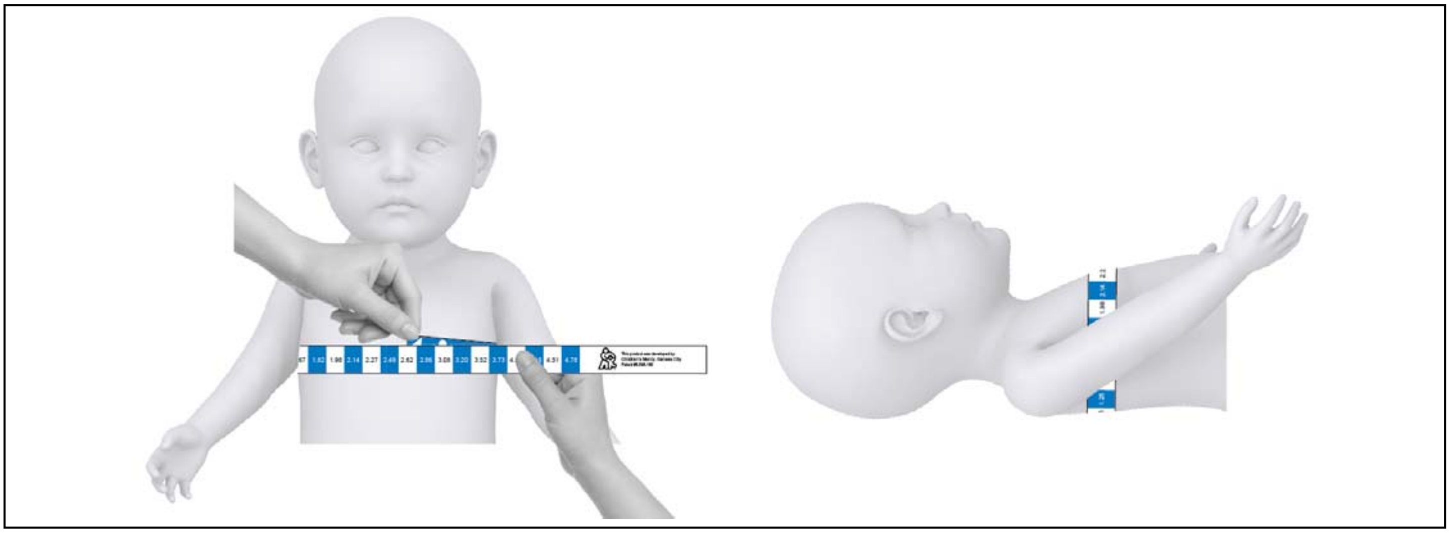

Copyright © 2018, Children's Mercy Hospital. All Rights Reserved.

Mercy babyTAPE - Instructions for Use

B. Determining the Weight

Add the number identified when measuring head circumference and chest circumference together. This is the infant's estimated weight in kilograms. You may use the table provided to verify your addition

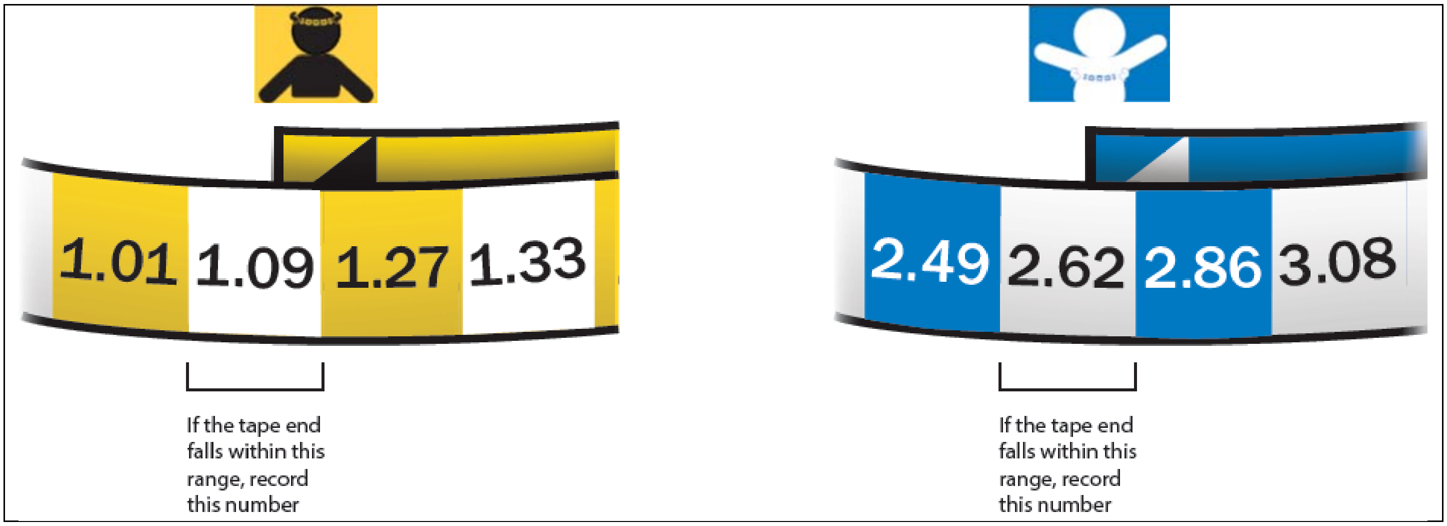

Copyright © 2018, Children's Mercy Hospital. All Rights Reserved.
