# Supplementary figures and images for: Validation and human factor analysis study of an infant weight estimation device
Source: BMC Pediatr. 2020 Jan 22;20:30. doi: 10.1186/s12887-020-1933-5 (PMC6977278; doi:10.1186/s12887-020-1933-5)

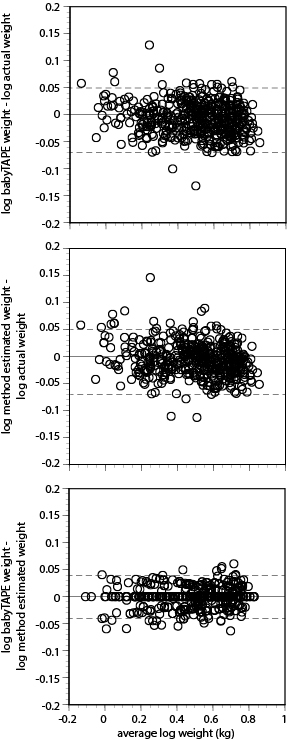

Supplement: Supplementary file 2 — Additional file 2. Supplemental Figure, Bland-Altman plots depicting the difference between device-predicted weight versus actual weight (upper), method-predicted weight versus actual weight (middle), and device-predicted weight versus method-predicted weight (lower). Dashed lines depict the 95% limits of agreement. [file 12887_2020_1933_MOESM2_ESM.jpg]
